# Breath Formate Is a Marker of Airway *S*-Nitrosothiol Depletion in Severe Asthma

**DOI:** 10.1371/journal.pone.0011919

**Published:** 2010-07-30

**Authors:** Roby Greenwald, Anne M. Fitzpatrick, Benjamin Gaston, Nadzeya V. Marozkina, Serpil Erzurum, W. Gerald Teague

**Affiliations:** 1 Department of Environmental Health, Rollins School of Public Health, Emory University, Atlanta, Georgia, United States of America; 2 Department of Pediatrics, Emory University School of Medicine, Atlanta, Georgia, United States of America; 3 Department of Pediatrics, University of Virginia, Charlottesville, Virginia, United States of America; 4 Department of Medicine and Pathobiology, Cleveland Clinic, Cleveland, Ohio, United States of America; University of Giessen Lung Center, Germany

## Abstract

**Background:**

Children with severe asthma have poor symptom control and elevated markers of airway oxidative and nitrosative stress. Paradoxically, they have decreased airway levels of *S*-nitrosothiols (SNOs), a class of endogenous airway smooth muscle relaxants. This deficiency results from increased activity of an enzyme that both reduces SNOs to ammonia and oxidizes formaldehyde to formic acid, a volatile carboxylic acid that is more easily detected in exhaled breath condensate (EBC) than SNOs. We therefore hypothesize that depletion of airway SNOs is related to asthma pathology, and breath formate concentration may be a proxy measure of SNO catabolism.

**Methods and Findings:**

We collected EBC samples from children and adolescents, including 38 with severe asthma, 46 with mild-to-moderate asthma and 16 healthy adolescent controls, and the concentration of ionic constituents was quantified using ion chromatography. The concentrations of EBC components with volatile conjugates were log-normally distributed. Formate was the principal ion that displayed a significant difference between asthma status classifications. The mean EBC formate concentration was 40% higher in samples collected from all asthmatics than from healthy controls (mean = 5.7 µM, mean±standard deviation = 3.1−10.3 µM vs. 4.0, 2.8−5.8 µM, p = 0.05). EBC formate was higher in severe asthmatics than in mild-to-moderate asthmatics (6.8, 3.7−12.3 µM vs. 4.9, 2.8−8.7 µM, p = 0.012). In addition, formate concentration was negatively correlated with methacholine PC_20_ (r = −0.39, p = 0.002, asthmatics only), and positively correlated with the NO-derived ion nitrite (r = 0.46, p<0.0001) as well as with total serum IgE (r = 0.28, p = 0.016, asthmatics only). Furthermore, formate was not significantly correlated with other volatile organic acids nor with inhaled corticosteroid dose.

**Conclusions:**

We conclude that EBC formate concentration is significantly higher in the breath of children with asthma than in those without asthma. In addition, amongst asthmatics, formate is elevated in the breath of those with severe asthma compared to those with mild-to-moderate asthma. We suggest that this difference is related to asthma pathology and may be a product of increased catabolism of endogenous *S*-nitrosothiols.

## Introduction

Individuals with severe asthma have poor symptom control despite treatment with high doses of inhaled and systemic corticosteroids [1]. In association with co-investigators involved in the NIH/NHLBI Severe Asthma Research Program (SARP), we have focused on the identification of clinical features and biomarkers that differentiate severe from mild-to-moderate asthma in childhood. Unlike their adult counterparts, children with severe asthma are more atopic, have relatively mild airflow limitation at baseline, and have a high degree of bronchodilator responsiveness compared to children with mild-to-moderate asthma [2]. These findings are associated with airway biochemical redox disturbances, characterized by a shift in the balance of airway glutathione from the reduced to the oxidized form, and increased concentrations of the oxidant biomarkers H_2_O_2_, 8-isoprostane, and malondialdehyde in airway surface liquid [3]. Furthermore, nitric oxide production is altered in a way that supports the enrichment of toxic nitrogen oxides [4]. Consequently, there is a relatively high level of oxidative and nitrosative stress in the airways of children with severe asthma as compared to those with mild-to-moderate asthma.

The difficulty of sampling airway biochemical markers in humans presents a significant challenge to the evaluation of airway status. Collection of bronchoalveolar lavage fluid and tracheal aspirate has previously been used to determine the concentration of *S*-nitrosothiols and other biomarkers in the airways [3], [5], [6], [7]. Unfortunately, the invasive nature of these procedures makes them less amenable in pediatric subjects and renders them unsuitable for large clinical studies. On the other hand, the collection of exhaled breath condensate (EBC) is a useful method to non-invasively sample airway lining fluids. EBC contains minute quantities of non-volatile material entrained from collapsed menisci of lower airway surface liquids [8], [9], [10] as well as volatile species that are the product of gas-aqueous phase partitioning anywhere along an exhaled breath flow path [11]. We have used ion chromatography to quantify the concentration of inorganic and low molecular weight organic ions in EBC [12], including both volatile and non-volatile species. This technique yields highly reproducible results with a detection limit in the tens to hundreds of nanomoles per liter. The purpose of the current study was to quantify the volatile and non-volatile ionic components of EBC of children enrolled in SARP and to identify ionic constituents that best correlate with clinical characteristics and established biomarkers. Furthermore, we examined the possibility that individual ions or associations of ions detected in EBC could be used to predict asthma severity.

## Methods

### Study sample and recruitment

This observational cross-sectional study was conducted based on the characterization procedures developed by the NIH/NHLBI Severe Asthma Research Program (SARP). The protocol was approved by the Emory University Institutional Review Board, and all procedures were monitored by an independent Data Safety Monitoring Board. The legal guardians of enrollees provided written informed consent, and the enrollees themselves gave assent when appropriate. All SARP participants met criteria for asthma as developed by the American Thoracic Society Guidelines.

To compare the ionic composition of EBC from asthmatic children to that of non-asthmatic children, we analyzed EBC samples collected from adolescent athletes enrolled in a previous study. The features of this control group and study methods used have been previously described [12], [13]. Briefly, an EBC sample was collected from each subject before and after outdoor exercise on ten different days in late summer in Atlanta. The age range of control subjects was 14–17 years while that of asthmatics was 6–17 years; however, the distribution of EBC formate concentration in asthmatics 6–13 years was essentially the same as that of asthmatics 14–17 years, suggesting that in this age range at least, age does not have a significant influence on EBC formate concentration. Since each participant in the control group provided up to 20 repeated measures of EBC, we selected at random one sample from each participant for our analyses. Control group samples were collected both before and after exercise, although the previous study did not find any influence of exercise on EBC formate [12]. In addition, analysis of the control group repeated measures indicated that the same-subject coefficient of variation for EBC formate was 33% over the course of the two week sampling period.

### Characterization procedures

SARP enrollees were designated as having either severe or mild-to-moderate asthma based on the definition developed by the American Thoracic Society Consensus Group (ATS Consensus on Severe Asthma). This characterization procedure has been previously described in detail [1] and has been validated by our group for use with pediatric subjects [2]. Severe asthma is defined by one or more major criteria (treatment with oral corticosteroids for at least 50% of the year prior to enrollment and/or a high daily dose of inhaled corticosteroids) and at least two minor criteria (treatment with a second daily controller medication, daily use of a short-acting bronchodilator, FEV_1_<80% predicted at baseline, more than one emergency department visit for asthma in the previous year, more than three oral corticosteroid bursts in the previous year, deterioration following a reduction in corticosteroid dose, or history of an asthma event requiring intubation). Participants who did not meet this definition of severe asthma were classified as having mild-to-moderate asthma.

### Sample collection and analysis

EBC was collected using the R-tube® (Respiratory Research, Charlottesville, VA). EBC pH was measured both before and after deaeration with argon. A separate aliquot was analyzed by ion chromatography using a previously described method [12]. Since the concentrations of ionic species with volatile conjugates are log-normally distributed, analyses of EBC constituents were performed using log-transformed values. The mean and spread of log-transformed variables were subsequently reverted to units of micromolar concentration for readability purposes, but note that the resulting ranges of mean±standard deviation are not symmetric with respect to the mean on a linear scale. We used SAS® software (Version 9.2, SAS Institute Inc., Cary, NC) to conduct principal component analysis of EBC constituents, bivariate linear correlations between each EBC component with all others and with individual clinical indicators related to asthma severity, and Student's t-tests to determine significant differences between the asthma severity classes.

## Results

### Subject characterization

The study sample included 38 children with severe asthma, and 46 children with mild-to-moderate asthma as well as 16 healthy adolescents enrolled in a previous study (**Table 1**). Compared to children with mild-to-moderate asthma, children with severe asthma were more likely to be self-identified as African-American, were treated with higher doses of inhaled corticosteroids, and were more likely to have been admitted to the hospital in the past. Likewise children with severe asthma had relatively reduced lung function, including lower baseline FEV_1_ percent predicted, lower FEV_1_/FVC, and air trapping as indicated by a higher RV/TLC ratio. Although children with severe asthma exhibited mild airflow obstruction at baseline, FEV_1_ percent predicted increased significantly following maximum bronchodilation, and post-bronchodilator spirometric variables in children with severe asthma were not significantly different from those of children with mild-to-moderate asthma. Children with severe asthma were more atopic, with higher serum IgE and a greater number of positive skin prick tests to aeroallergens.

**Table 1:** Characteristics of study participants^1^.

		severe (N = 38)		mild-to-moderate (N = 46)		non-asthmatic (N = 16)	
Age in years		11	(6–17)	10	(6–16)	15	(14–17)
Race:	African American	28	(74)	20	(43)	5	(31)
	Non-Hispanic white	7	(18)	20	(43)	11	(69)
	other	3	(8)	6	(13)		
Gender:	male	19	(50)	30	(65)	9	(56)
	female	19	(50)	15	(35)	7	(44)
BMI		20.4	(13.7−38.0)	20.3	(13.2−40.6)	19.8	(17.5−23.5)
ICS dose (µg fluticasone equivalents/day)^2^		853	(176−1000)	352	(0−1000)	0	(0)
Serum IgE (IU/mL)^2^		378	(4−5458)	142	(2−3484)	not measured	
Pulmonary function						not measured	
FVC (% predicted)		96	(65−128)	103	(77−141)		
FEV_1_ (% predicted)		82	(48−114)	97	(64−123)		
FEV_1_/FVC		0.74	(0.59−0.96)	0.82	(0.56−1.00)		
FEF_25–75_ (% predicted)		67	(26−193)	95	(32−234)		
ΔFEV_1_ post-bronchodilator		15%	(−1.4−55%)	7.8%	(−5−33%)		
RV/TLC		0.34	(0.16−0.86)	0.24	(0.14−0.39)	not measured	
Methacholine PC_20_		1.11	(0.14−20)	5.26	(0.09−20)	not measured	

1 Values are either frequency (percentage) or mean (range).

2 Data were logarithmically transformed prior to analysis.

### Principal component analysis and bivariate correlations

Nearly all EBC samples contained quantifiable concentrations of the anions lactate, acetate, propionate, formate, butyrate, pyruvate, chloride, nitrite, nitrate, sulfate, and oxalate as well as the cations sodium, ammonium, potassium, magnesium, and calcium. The concentration of ammonium was approximately an order of magnitude greater than other measured ions.

**Figure 1:**
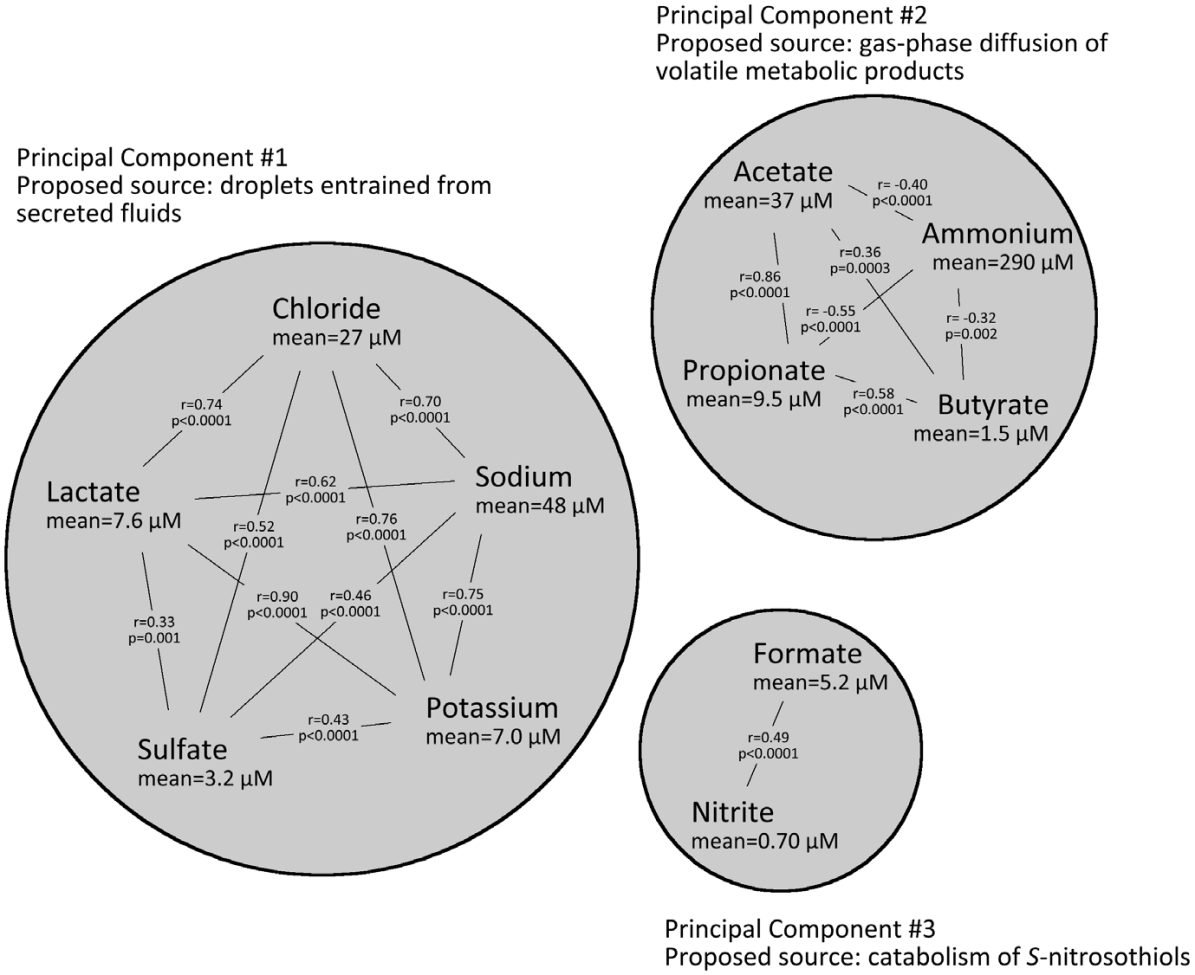
Principal Component Analysis of EBC collected from all subjects. Each cluster is scaled according to the fraction of the total variance explained by that principal component. The Pearson correlation coefficients are given between all species within each cluster.

Principal component analysis identified three clusters of EBC constituents that account for most of the variance in ionic composition (**Figure 1**). The first principal component, accounting for 32% of the total variance, was closely correlated (p<0.0001) with the non-volatile species lactate (r = 0.92), chloride (r = 0.81), sulfate (r = 0.36), sodium (r = 0.82) and potassium (r = 0.95). The second component, accounting for 22% of the variance, was correlated (p<0.0001) with species with a volatile conjugate such as acetate (r = 0.79), propionate (r = 0.90), and butyrate (r = 0.78) and was inversely correlated with ammonium (r = −0.59). The third principal component, accounting for 14% of the variance, was correlated (p<0.0001) with formate (r = 0.62) and nitrite (r = 0.84). These three clusters likely represent groups of ions with similar sources: non-volatile ions associated with fluid secretion (chloride, sodium, potassium), volatile organics associated with metabolic processes (acetate, propionate, ammonium) and volatile species with a source distinct from other volatiles, perhaps related to nitrosylative processes (formate, nitrite).

Examination of the bivariate correlations between ions revealed a similar pattern: many non-volatile species were correlated with one other as were several organic species with a volatile conjugate, namely acetate, propionate, and butyrate. Ammonium was inversely correlated with these acidic species but did not otherwise exhibit a relationship with any other constituent. In addition, formate was significantly correlated with two clinical variables (not measured in controls), namely the methacholine PC_20_ (for log-transformed formate, r = −0.39, p = 0.002) and serum IgE (r = 0.28, p = 0.016).

### Differences between asthma severity classifications

**Figure 2:**
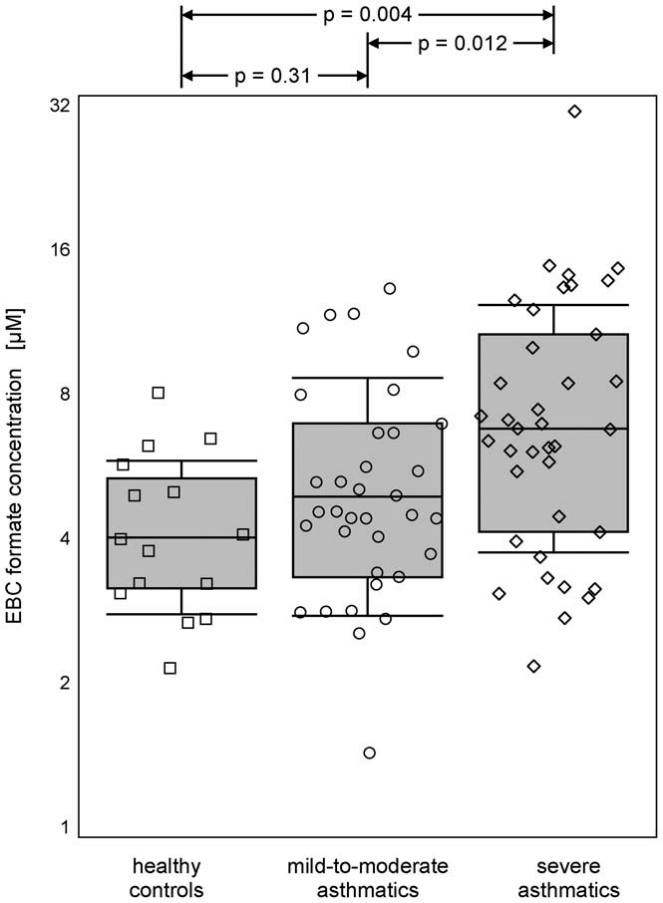
The distributions of EBC formate concentration for healthy adolescents and children with mild-to-moderate or severe asthma. Markers represent individual measurements; boxes represent the 25^th^, 50^th^, and 75^th^ percentiles; whiskers represent the mean±standard deviation.

The key differences in EBC ionic composition between asthma severity classifications were found to be formate and to a lesser extent, nitrite concentration. The mean EBC formate concentration was 40% higher in samples collected from all asthmatics than from healthy controls (mean = 5.7 µM, mean±standard deviation = 3.1−10.3 µM vs. 4.0, 2.8−5.8 µM, p = 0.05). In addition, EBC formate was significantly higher in severe asthmatics than in mild-to-moderate asthmatics (**Figure 2**). EBC nitrite likewise displayed a statistically-significant difference between asthmatic subjects and healthy controls (0.92, 0.37−2.3 µM vs. 0.24, 0.13−0.44 µM, p<0.0001), though the difference between severe and mild-to-moderate asthmatics was modest and not significant (1.0, 0.43−2.3 µM vs. 0.86, 0.33−2.3 µM, p = 0.45). Other EBC ions either did not display a difference between groups and/or the difference was not significant.

## Discussion

Of the ionic components measured in EBC collected from asthmatic and healthy children and adolescents, formate displayed the most notable difference between asthma severity classifications. Principal component analysis suggests that the source of formate in EBC is distinct from most other measured ionic species. Despite its high volatility in the non-ionized state, formate is not highly correlated with other volatile organic acids commonly detected in EBC. In addition, EBC formate is associated with the NO-derived ion nitrite, which itself displays more modest stratification by asthma severity. Furthermore, EBC formate was significantly correlated with clinical variables associated with asthma severity, namely methacholine PC_20_ and serum IgE. Given the strength of the significant difference in EBC formate between asthma severity classifications, we suggest that the higher formate seen in the breath condensate of severe asthmatics may be related to asthma pathology.

A potential mechanism that conceptually explains both elevated breath formate levels and their association with asthma severity involves the catabolism of *S*-nitrosothiols (SNOs). SNOs are a novel class of endogenous bronchodilators [6] that are formed by the addition of nitrosonium to peptide or protein sulfhydryl group s[14], [15], [16], [17]. These compounds serve as a stable reservoir of nitrosonium [18], [19] and play a vital role both in airway smooth muscle relaxation and in prevention of tachyphylaxis of the β_2_ adrenoreceptor. The principal SNO in healthy human airways is *S*-nitrosoglutathione (GSNO) [6]. GSNO activity is regulated by an alcohol dehydrogenase enzyme [20], [21], [22], [23] referred to in this context as GSNO-reductase (GSNOR). In the presence of NADH, GSNOR reduces GSNO to form glutathione sulfinamide [22]; however, GSNOR also oxidizes the formaldehyde-glutathione complex *S*-hydroxymethylglutathione to form *S*-formylglutathione [22], [24]. *S*-formylglutathione is in turn hydrolyzed to glutathione and formate [25]. Thus an increase in GSNOR activity would be expected to lead to both a loss of GSNO [24], [26] and an increase in formate production.

A growing body of evidence suggests that GSNO dysregulation is intimately involved in the pathology of asthma. Analysis of fluids obtained from the airways of children with respiratory failure and severe asthma have been found to have lower levels of SNOs relative to healthy children [7]. This depletion is likely related to increased GSNOR activity. Que *et al.* [27] have shown that mice deficient in GSNOR have high levels of airway GSNO and are protected from ovalbumin-induced airway hyperresponsiveness to methacholine whereas hyperresponsive mice have high levels of GSNOR in their airways. This same group subsequently demonstrated that GSNOR activity is higher in asthmatic humans than in controls and is inversely correlated with both airway *S*-nitrosothiol content and methacholine PC_20_ [28]. Whalen *et al*. [29] found that GSNOR prevents *S*-nitrosylation of G-protein-coupled receptor kinase 2 and that GSNOR deficient mice are protected from tachyphylaxis to β_2_ agonists. We therefore hypothesize that SNO degradation perturbs airway tone in asthma and suggest that GSNO may play an important role in future therapeutic strategies for the treatment of asthma.

Unfortunately, the concentrations of SNOs and GSNOR in human airways have proven difficult to evaluate. The *S*-nitrosothiol bond is notoriously labile, introducing the potential for false positives and negatives [30], and in addition, SNO concentrations in the airways of asthmatics are extremely low, indeed, nearly undetectable in samples of airway fluid. This problem is compounded by high levels of protons and nitrite in asthmatic airways [6], [31], each of which can artifactually increase measured SNO levels. Formic acid on the other hand is a volatile species related to *S*-nitrosothiol catabolism and is easily detectable as formate in EBC with no sample preparation needed [12]. For non-volatile compounds, the variable degree of dilution with water vapor can present a challenge to proper interpretation of EBC concentration data [32], [33]; however, for volatile compounds such as formic acid, EBC concentration is influenced by factors related to volatilization more so than water vapor condensation. For example, the fraction of formic acid in epithelial lining fluid that is volatile is pH-dependent (0.55% is volatile at pH = 6.0, 0.17% at pH = 6.5, and 0.055% at pH = 7.0), and in addition, volatilization from non-airway sources could contribute to EBC formate concentration. Furthermore, the concentration of the *S*-hydroxymethylglutathione substrate is related to formaldehyde exposure [24]; hence formaldehyde exposure would be expected to increase EBC formate. If analysis of EBC formate concentration is indeed a valid proxy for GSNOR activity, EBC formate might be used as a non-invasive measure to identify asthmatic patients at risk for GSNO depletion to receive therapies designed to enhance or replace endogenous GSNO levels.
